# Influences of Extracellular Polymeric Substances on the Dewaterability of Sewage Sludge during Bioleaching

**DOI:** 10.1371/journal.pone.0102688

**Published:** 2014-07-22

**Authors:** Jun Zhou, Guanyu Zheng, Xueying Zhang, Lixiang Zhou

**Affiliations:** 1 Department of Environmental Engineering, College of Resources and Environmental Sciences, Nanjing Agricultural University, Nanjing, China; 2 College of Biotechnology and Pharmaceutical Engineering, Nanjing Tech University, Nanjing, China; 3 Jiangsu Collaborative Innovation Center for Solid Organic Waste Resource Utilization, Nanjing, China; 4 College of Environment, Nanjing Tech University, Nanjing, China; Loyola University Medical Center, United States of America

## Abstract

Extracellular polymeric substances (EPS) play important roles in regulating the dewaterability of sludge. This study sought to elucidate the influence of EPS on the dewaterability of sludge during bioleaching process. Results showed that, in bioleaching system with the co-inoculation of *Acidithiobacillus thiooxidans* TS6 and *Acidithiobacillus ferrooxidans* LX5 (*A. t*+*A. f* system), the capillary suction time (CST) of sludge reduced from 255.9 s to 25.45 s within 48 h, which was obviously better than the controls. The correlation analysis between sludge CST and sludge EPS revealed that the sludge EPS significantly impacted the dewaterability of sludge. Sludge CST had correlation with protein content in slime and both protein and polysaccharide contents in TB-EPS and Slime+LB+TB layers, and the decrease of protein content in slime and decreases of both protein and polysaccharide contents in TB-EPS and Slime+LB+TB layers improved sludge dewaterability during sludge bioleaching process. Moreover, the low sludge pH (2.92) and the increasing distribution of Fe in the solid phase were another two factors responsible for the improvement of sludge dewaterability during bioleaching. This study suggested that during sludge bioleaching the growth of *Acidithiobacillus* species resulted in the decrease of sludge pH, the increasing distribution of Fe in the solid phase, and the decrease of EPS content (mainly including protein and/or polysaccharide) in the slime, TB-EPS, and Slime+LB+TB layers, all of which are helpful for sludge dewaterability enhancement.

## Introduction

Activated sludge method is an effective way of treating wastewater, but it has a serious drawback of producing huge amounts of excess waste sludge. Thus, a subsequent dewatering step is usually needed to reduce the sludge volume for facilitating their transport and handling and to minimize the addition of bulking agents during composting or the energy needed in case of drying or incineration [1]–[3]. The moisture content of sewage sludge can only be reduced to around 70% when it is treated by mechanical dewatering methods [4]–[6] because of the extremely low dewaterability of sewage sludge, and the associated capital and operating costs usually account for as high as 25–50% of the total expenses of the whole wastewater treatment processes [4], [7]. Therefore, effective and economical methods should be developed for treating the huge amount of sludge with the aim of enhancing sludge dewaterability, which usually refers to the degree of difficulty in removing water from sludges.

Previous studies have revealed that the dewaterability of sludge is controlled by many factors, such as sludge particle size, flocs structure and extracellular polymeric substances (EPS) in sludge [7]–[8]. Among these, sludge EPS plays a key role [9]–[11]. EPS is a major component of sludge floc matrix, and the presence of a large amount of EPS is the main reason for the difficulty in sludge dewatering [9]–[11]. Polysaccharide, protein and DNA, which entrap water and have high viscosities, are the compositions of sludge EPS [11]–[13]. Many studies focused on the influence of sludge EPS on the thickening and dewatering of sludge [12]–[14]. Chen et al [1] found that sludge dewaterability would be improved after EPS was removed. However, some studies showed that sludge dewaterability improved when the EPS content increased [11], [15]. Houghton et al [16] proposed that the dewaterability of sludge initially increased with the raise of EPS content, but then decreased once the EPS content exceeded a certain threshold. It was also reported that the compositions and properties of EPS, rather than their quantities, were more important in governing sludge dewaterability [9], [17], and various components in sludge EPS possessed different effects in sludge dewatering [11], [18]. For instances, some researchers found that proteins had a high water-holding capacity, and reducing protein fraction or increasing the content of polysaccharide fraction in sludge EPS could improve sludge dewaterability [15], [19]. In addition, a concept of sludge flocs stratification was introduced by Yu et al., who found that EPS in sludge flocs were consisted of slime, loosely bound EPS (LB-EPS) and tightly bound EPS (TB-EPS) [18]. Furthermore, the increase of protein content or the content ratio of protein to polysaccharide (PN/PS) in slime had a negative effect on the sludge dewaterability improvement [12]–[13]. Therefore, there are still some controversies in the relationship between sludge EPS and its dewaterability.

Bioleaching technique, which was successfully applied in biohydrometallurgy for extracting metals from sulfide minerals [20]–[21], has been developed in the last decade as an attractive method of removing metals from sewage sludge [22]–[24]. During bioleaching, bio-oxidation of S^0^ by *Acidithiobacillus thiooxidans* and/or the hydrolysis of Fe^3+^ driven from the bio-oxidation of Fe^2+^ by *Acidithiobacillus ferrooxidans* produce H^+^ that causes the decrease of sludge pH, and the low sludge pH and the dominant *Acidithiobacillus* species are two main characteristics of sludge bioleaching system [25]. It was reported recently that bioleaching can not only remove heavy metals from sludge matrix, but also improve sludge dewaterability by 4–10 times [26]. The moisture content of dewatered bioleached sludge cake is as low as 60% in pilot-scale studies using bioleaching conditioning and diaphragm filter presses [27], [28]. However, to date, it is still unclear why the sludge dewaterability can be drastically improved by bioleaching process, and how the growth of *Acidithiobacillus* species influences the content and composition of sludge EPS during bioleaching treatment.

Therefore, the objectives of the present study are to (1) explore the variation of sludge EPS during sludge bioleaching, and (2) elucidate the relationship between the content and composition of sludge EPS and the sludge dewaterability during bioleaching process. Understanding the detailed relationship between sludge EPS and sludge dewaterability is helpful for further optimizing sludge bioleaching conditions to improve sludge dewaterability.

## Materials and Methods

### Ethics statement

No specific permits were required for the described field studies and no specific permissions were required for these locations. The location is not privately-owned or protected in any way.

### Sludge sample

Waste activated sludge was collected from Lucun Wastewater Treatment Plant, Wuxi City, China, and stored at 4 °C until use. The pH value of sludge was measured immediately after collection, and the solid content was measured by oven-drying at 105°C. Organic matter content and volatile suspend solids (VSS) were determined according to their standard methods, and dried sludge samples were first digested and then measured for total N and P [29]. Selected physicochemical properties of sludge are shown in Table 1.

**Table 1:** Selected physicochemical characteristics of sludge.

pH	Solid content (%)	VSS (%)	Total N (%)	Total P (%)	EC (ms/cm)	CST (s)
6.97	2.25	73.1	6.19	2.79	5.80	255.9

### Microorganisms and bioleaching inoculum preparation

*Acidithiobacillus ferrooxidans* LX5 (CGMCC No. 0727) and *Acidithiobacillus thiooxidans* TS6 (CGMCC No. 0759) obtained from China General Microbiological Culture Collection Center (CGMCC) were cultivated in modified 9K and SM liquid medium [25], [30], respectively. The modified 9K and SM mediums autoclaved at 121°C for 15 min were adjusted to pH 2.5 and 3.0 with sulfuric acid, and then spiked with 44.2 g/L of 0.22 µm membrane-filtered FeSO_4_·7H_2_O or 10 g/L of elemental sulfur (S^0^) as the energy source, respectively [25], [30]. These bacteria were cultured in 500 mL Erlenmeyer flasks each containing 250 mL of modified 9K or SM medium on a gyratory shaker at 200 rpm and 28°C for 3–4 days until their respective cell densities reached around 10^8^ cells/mL.

The bioleaching inoculum was prepared by mixing 15 mL of *A. ferrooxidans* and 15 mL of *A. thiooxidans* in 500 mL Erlenmeyer flasks each containing 270 mL of sludge supplemented with 2 g/L of Fe^2+^ and 1 g/L of S^0^. Then all flasks were agitated on a gyratory shaker at 28°C and 180 rpm until sludge pH dropped to 2.0, which is usually considered as the terminal stage of sludge bioleaching [24]. The bioleached sludge was employed as bioleaching inoculum in the following experiments. The counts of *A. ferrooxidans* LX5 and *A. thiooxidans* TS6 in this bioleaching inoculum were 1.74×10^8^ CFU/g dw and 2.68×10^8^ CFU/g dw, respectively.

### Bioleaching experiment

The bioleaching experiment was conducted in a series of 500 mL Erlenmeyer flasks, each containing 270 mL fresh sludge followed by the addition of (1) 30 mL autoclaved bioleaching inoculum (control 1, hereinafter named by CK1), (2) 2 g/L Fe^2+^+1 g/L S^0^+30 mL autoclaved bioleaching inoculum (control 2, hereinafter named by CK2), and (3) 2 g/L Fe^2+^+1 g/L S^0^+30 mL bioleaching inoculum (hereinafter named by the *A. t*+*A. f*). The molar ratios of 2 g/L Fe^2+^ and 1 g/L S^0^ were 0.036 mol/L Fe^2+^ and 0.031 mol/L S^0^, respectively. All flasks were incubated on a gyratory shaker at 28°C and 180 rpm. During the incubation, 8 mL of sludge sample was withdrawn from each flask at 12 h intervals and determined for sludge pH. Subsequently, these samples were centrifuged at 12, 000×g for 15 min, filtered through 0.45 µm membrane filter, and determined for Fe^2+^ and total Fe by using 1,10-phenanthroline method [31]. The distribution ratio of Fe in the solid phase of sludge to the aqueous phase of sludge can be calculated by using equation (1). Meanwhile, 15 mL sludge was also withdrawn from each flask at 12 h intervals and used for the extraction of sludge EPS. The dewaterability of sludge was measured as capillary suction time (CST) by using a capillary suction timer (Model 304 M, Triton, Britain). 
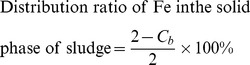
(1)(where 2 is the initial concentration of total Fe in the sludge, and *C_b_* is the concentration of total Fe in the centrifuged solution)

All treatments were done in triplicate throughout the present study unless otherwise noted, and data presented were the mean values of the triplicate samples with standard deviations.

### EPS extraction method

The sludge was centrifuged at 550 g for 15 min, and organic matters in the supernatant were the slime [18]. Then the sludge pellets were washed twice with 0.05% (*w*/*w*) NaCl solution. After that, the collected sludge pellets were resuspended by 0.05% (*w*/*w*) NaCl solution to its original volume. Ultrasound was performed on the suspension for 2 min, followed by the horizontal oscillation for 10 min, and then ultrasound was repeated again for 2 min. Thereafter, the LB-EPS were harvested by centrifugation at 9000×g and 4°C for 15 min. The residual sludge pellets were resuspended to the predetermined volume with 0.05% (*w*/*w*) NaCl solution, followed by the horizontal oscillation at 28°C and 180 rpm for 10 min. Afterward, the sludge was heated at 70°C for 30 min, and then the TB-EPS were harvested by centrifuging at 20, 000×g and 4°C for 20 min to remove remaining sludge components. The supernatants were the TB-EPS fraction. The 0.45 µm acetate cellulose membranes and the dialysis membranes of MWCO of 3500 Da (Shanghai Sangon Biotechnology, China) were used to remove the particulates and low-molecular-weight (MW) metabolites in the slime, LB-EPS and TB-EPS solutions before chemical analysis [18].

Polysaccharide content in the EPS solution was measured by using the anthrone method [32]. Protein content was measured by using the method proposed by Lowry et al. [33] and albumin was used as a standard solution [34]. PN/PS was the content ratio of protein to polysaccharide. The DNA content in EPS solutions was determined by using the diphenylamine colorimetric method [35], in which fish sperm deoxyribonucleic (DNA, Shanghai Sangon Biotechnology) was used as a standard solution.

## Results and Discussion

### Changes of sludge pH, concentrations of Fe^2+^ and total Fe, and sludge dewaterability during sludge bioleaching

**Figure 1:**
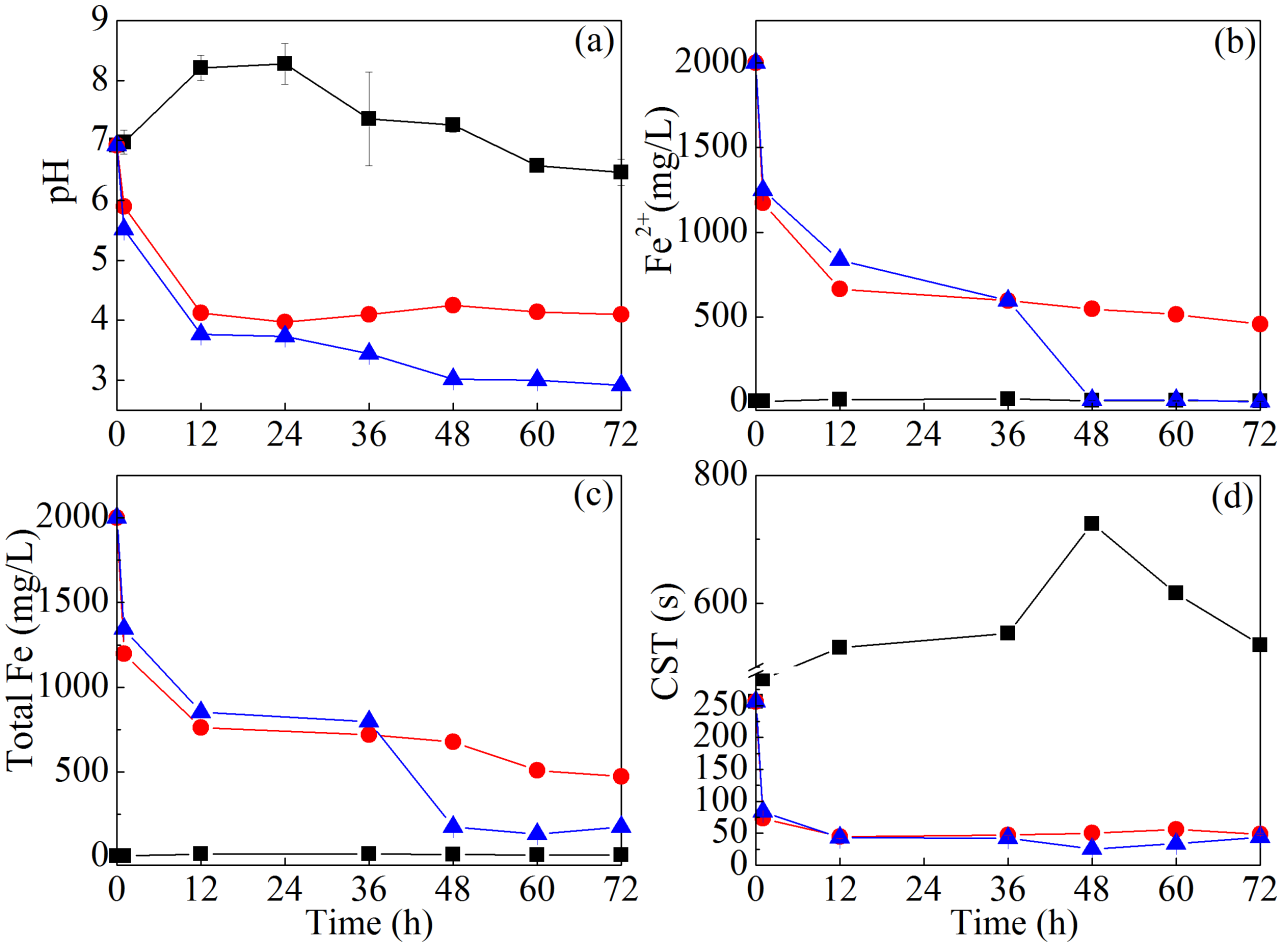
Changes of sludge pH, concentrations of Fe^2+^ and total Fe, and sludge CST during the sewage sludge bioleaching. (▪ CK1, ○CK2, △*A.t*+*A.f*).

It can be seen from Fig. 1a that, in the treatment inoculated with *A. thiooxidans* TS6 and *A. ferrooxidans* LX5 (*A. t*+*A. f*), the pH value of sludge decreased from 6.92 to 2.92 in the 72 h bioleaching period. However, in the CK1 system, which was without either the inoculation of these two *Acidithiobacillus* species or the addition of energy sources, sludge pH increased initially, decreased slowly and finally stabilized at pH 6.47 till the end of bioleaching. In the CK2 system, which was only added with Fe^2+^ and S^0^ as energy sources, sludge pH decreased from 6.92 to approximately 4.1 and then stabilized at this level until the end of bioleaching, most probably due to the hydrolysis of Fe^3+^ resulting from the chemical oxidation of Fe^2+^ by O_2_ in air [30].

Fig. 1b and Fig. 1c show the concentration changes of Fe^2+^ and total Fe during sludge bioleaching, respectively. The concentrations of Fe^2+^ and total Fe almost did not change in the CK1 system due to no addition of any energy source. Since Fe^2+^ is chemically unstable at neutral pH and can be oxidized to Fe^3+^, which is then easily precipitated as iron hydroxides or ferric hydroxysulfates and thus lowering sludge pH [30], [36], Fe^2+^ and total Fe concentrations in the CK2 system decreased to 458 mg/L and 472 mg/L, respectively, after the incubation. The concentration of Fe^2+^ in the *A. t*+*A. f* system decreased rapidly with the extension of reaction time, and the oxidation rate of Fe^2+^ reached about 100% within 48 h. The concentration change of total Fe in the *A. t*+*A. f* system was similar with that of Fe^2+^, and the decrease of total Fe concentration may be resulted from the formation of ferric hydroxide minerals and the adsorption of Fe^3+^ onto the surface of sludge granules during the bioleaching process [36].

Sludge CST is a widely used means of gauging sludge dewaterability [1], [13]. Fig. 1d displays the change of sludge CST during the bioleaching of sludge. Sludge CST gradually increased in the CK1 system and then had a slight decrease at the end of bioleaching, indicating that the dewaterability of sludge deteriorated in such pure aerobic digestion systems [37]. In the CK2 system, sludge CST was shortened from 255.9 s for fresh sludge to 44.35 s in 12 h and then gradually increased, and the decrease in sludge CST was most probably resulted from the coagulation effect of ferrous ion. The dewaterability of sludge in *A. t*+*A. f* system was much better than that of the two controls, as shown that sludge CST in *A. t*+*A. f* system decreased to only 25.45 s within 48 h. Therefore, the dewaterability of sludge was greatly improved by the bioleaching process. It is worthy to note that in bioleaching system when the solid content of sludge increases, the amount of energy substance (Fe^2+^or S^0^) added should be increased and the bioleaching time would be extended [22], [25]. The main characteristics of bioleaching system are low sludge pH and the primary *Acidithiobacillus* species in the bioleached sludge. Meanwhile, it is well known that EPS is regarded as the key factor in regulating the dewaterability of sludge [13]. Thus, the improvement of sludge dewaterability during bioleaching may result from the variation of sludge EPS induced by the low sludge pH and/or the growth of *Acidithiobacillus* species.

### Change of sludge EPS during bioleaching

**Figure 2:**
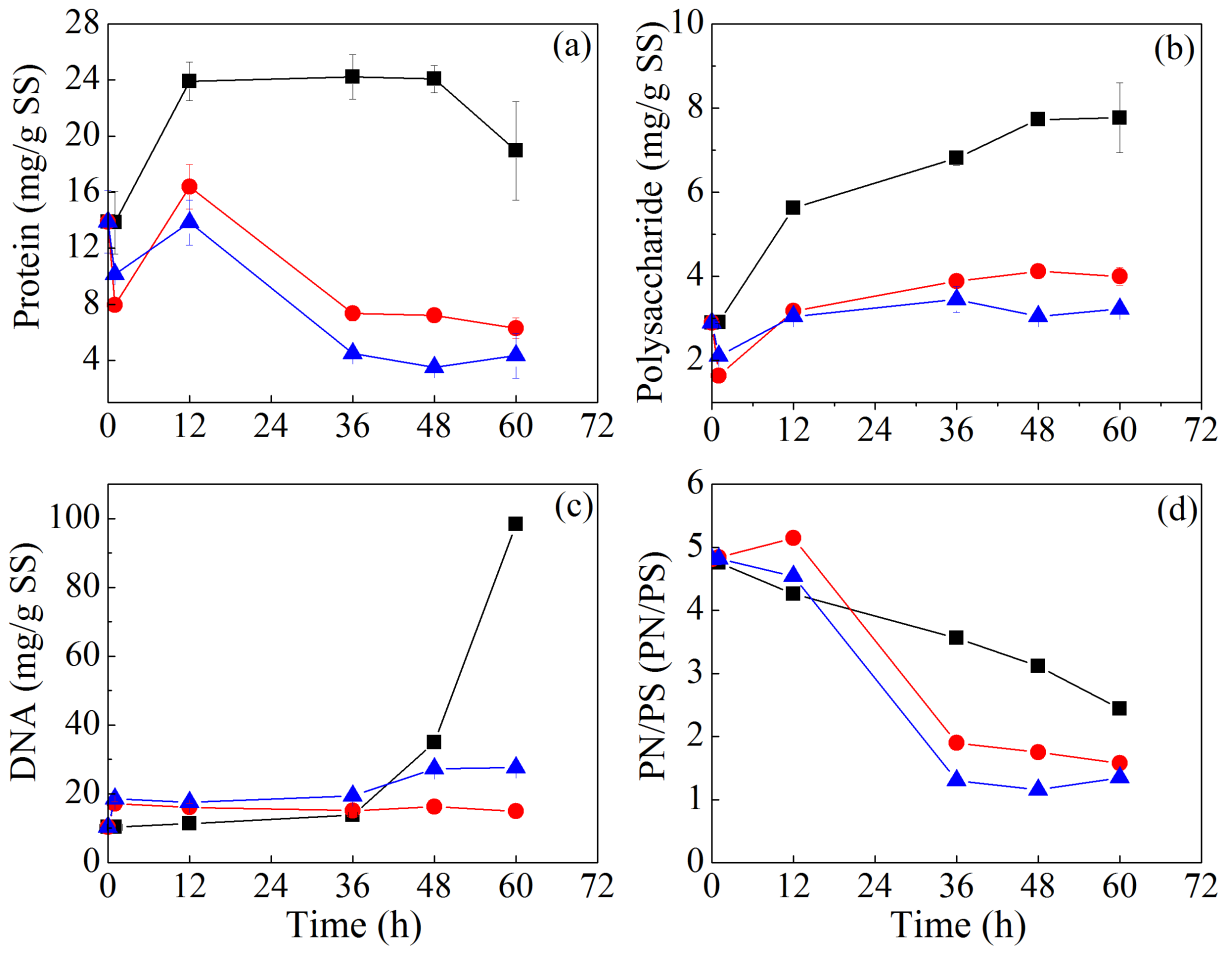
Variation of the content of protein (a), polysaccharide (b) and DNA (c), and PN/PS (d) in the slime during sludge bioleaching process. (PN/PS: protein/polysaccharide) (▪ CK1, ○CK2, △*A.t*+*A.f*).

Previous studies revealed that EPS plays an important role in regulating sludge dewaterability [13]. Fig. 2 presents the content changes of protein, polysaccharide and DNA in slime of EPS, and the content ratio of protein to polysaccharide (PN/PS) in slime during sludge bioleaching process. It was found that the contents of protein, polysaccharide and DNA were 13.89, 2.89 and 10.29 mg/g SS, respectively, and PN/PS was 4.81 in the slime of the original sludge. Protein, polysaccharide and DNA contents in slime gradually increased in CK1, implying that the EPS was released to the slime in such aerobic digestion process. Similar results were also found in other report [37], and the release of biopolymers to the slime would lead to the deterioration of sludge dewaterability. The content changes of protein, polysaccharide, DNA in slime layer in CK2 and *A. t*+*A. f* systems were different from that in CK1 system. The amount of protein in slime gradually decreased in CK2 and *A. t*+*A. f* systems, and protein in *A. t*+*A. f* system was always lower than that in CK2. Polysaccharide content in slime of CK2 and *A. t*+*A. f* systems had a slight increase, and the increase in CK2 was higher than that in the *A. t*+*A. f* system. DNA content in slime layer of CK1, CK2 and *A. t*+*A. f* systems all increased, while PN/PS gradually decreased and exhibited no differences in these two controls and one treatment systems.

**Figure 3:**
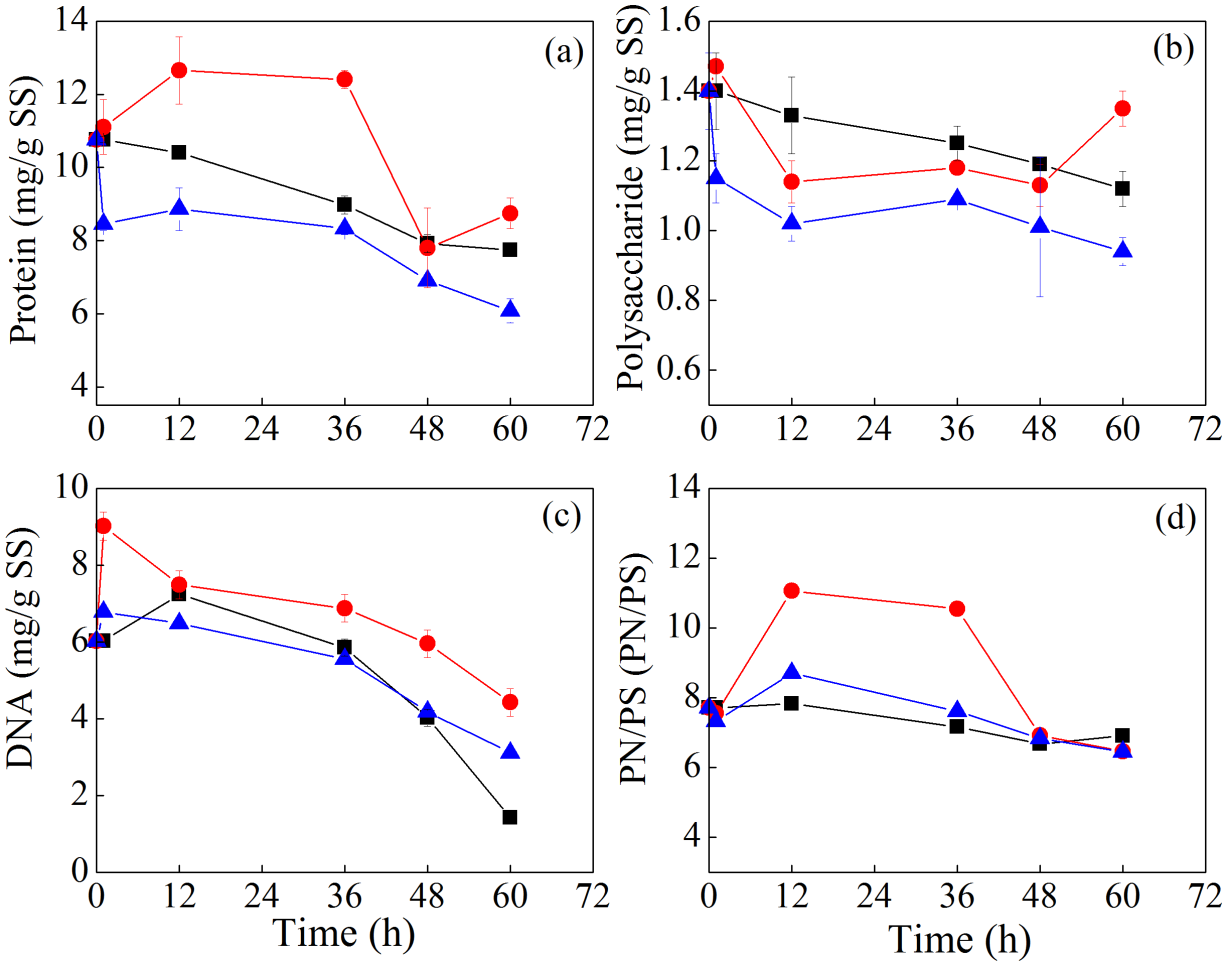
Variation of the content of protein (a), polysaccharide (b) and DNA (c), and PN/PS (d) in LB-EPS during sludge bioleaching process. (PN/PS: protein/polysaccharide) (▪ CK1, ○CK2, △*A.t*+*A.f*).

Fig. 3 presents the chemical composition change of LB-EPS during sludge bioleaching. In the LB-EPS of original sludge, the contents of protein, polysaccharide, DNA were 10.76, 1.40, 6.02 mg/g SS and the PN/PS was 7.71. Protein content in LB-EPS of CK2 had a slight increase initially and then gradually decreased, whereas it always decreased in the *A. t*+*A. f* system. The content of polysaccharide in LB-EPS of CK1, CK2, and *A. t*+*A. f* systems did not change during the treatment process, while the content of DNA in LB-EPS gradually decreased in CK1, CK2 and *A. t*+*A. f* systems and no obvious difference among these treatments was observed. PN/PS in LB-EPS of CK2 system increased during the treatment, and no obvious change of PN/PS in LB-EPS was observed in CK1 and *A. t*+*A. f* systems.

**Figure 4:**
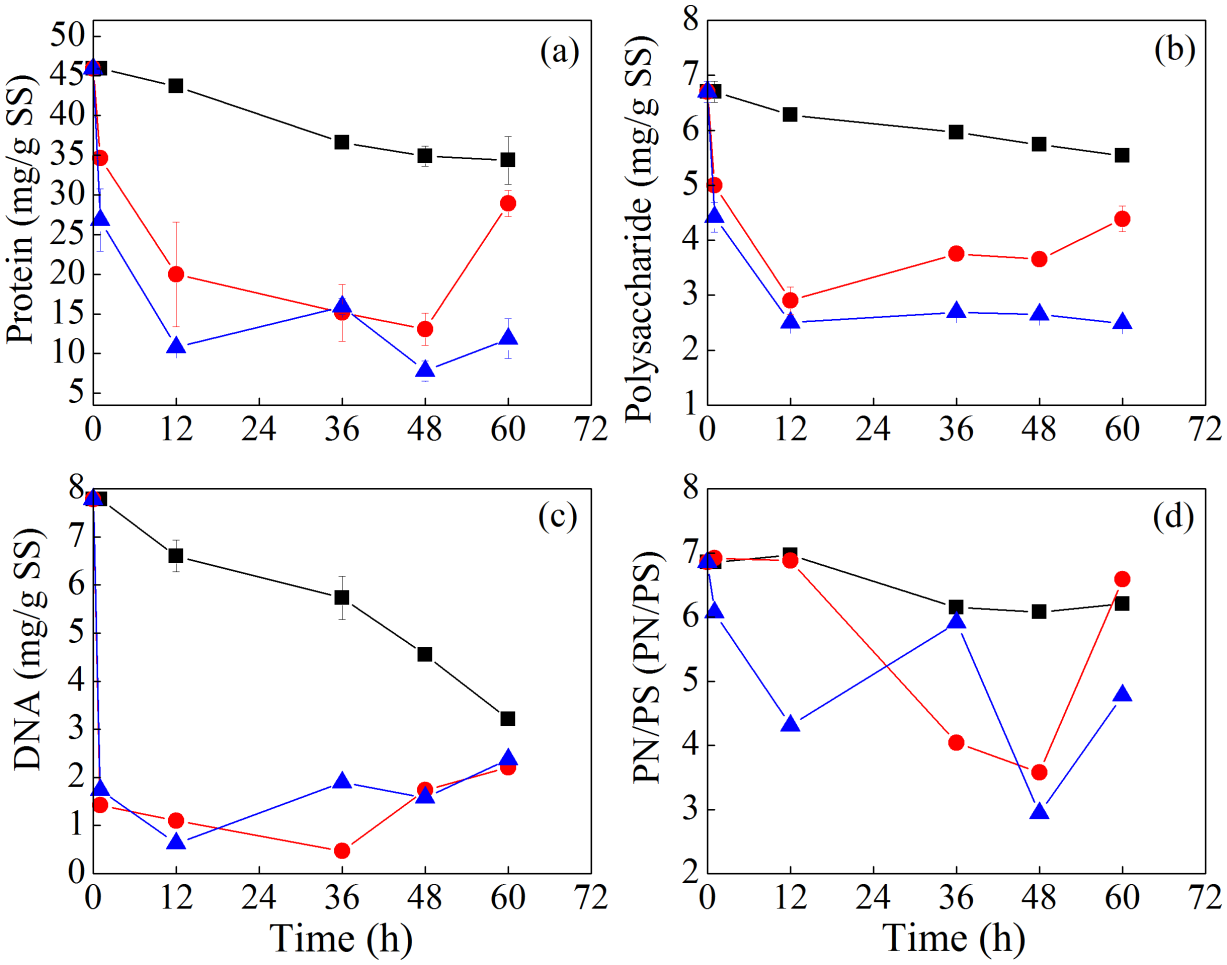
Variation of the content of protein (a), polysaccharide (b) and DNA (c), and PN/PS (d) in TB–EPS during sludge bioleaching process. (PN/PS: protein/polysaccharide) (▪ CK1, ○CK2, △*A.t*+*A.f*).

Fig. 4 displays the changes of chemical compositions of TB-EPS during sludge bioleaching. In the TB-EPS of original sludge, the contents of protein, polysaccharide, and DNA were 45.91, 6.70 and 7.78 mg/g SS, respectively, and PN/PS was 6.86. It was found that protein content in TB-EPS gradually decreased in all these systems during the incubation, and the protein content in TB-EPS of the *A. t*+*A. f* system decreased much more than that of the two controls. Polysaccharide content in TB-EPS of these three systems exhibited the similar trend as that of protein. DNA content and PN/PS in TB-EPS also decreased in all these systems, while there was a little increase of DNA content in CK2 and *A. t*+*A. f* systems at the end of treatment.

**Figure 5:**
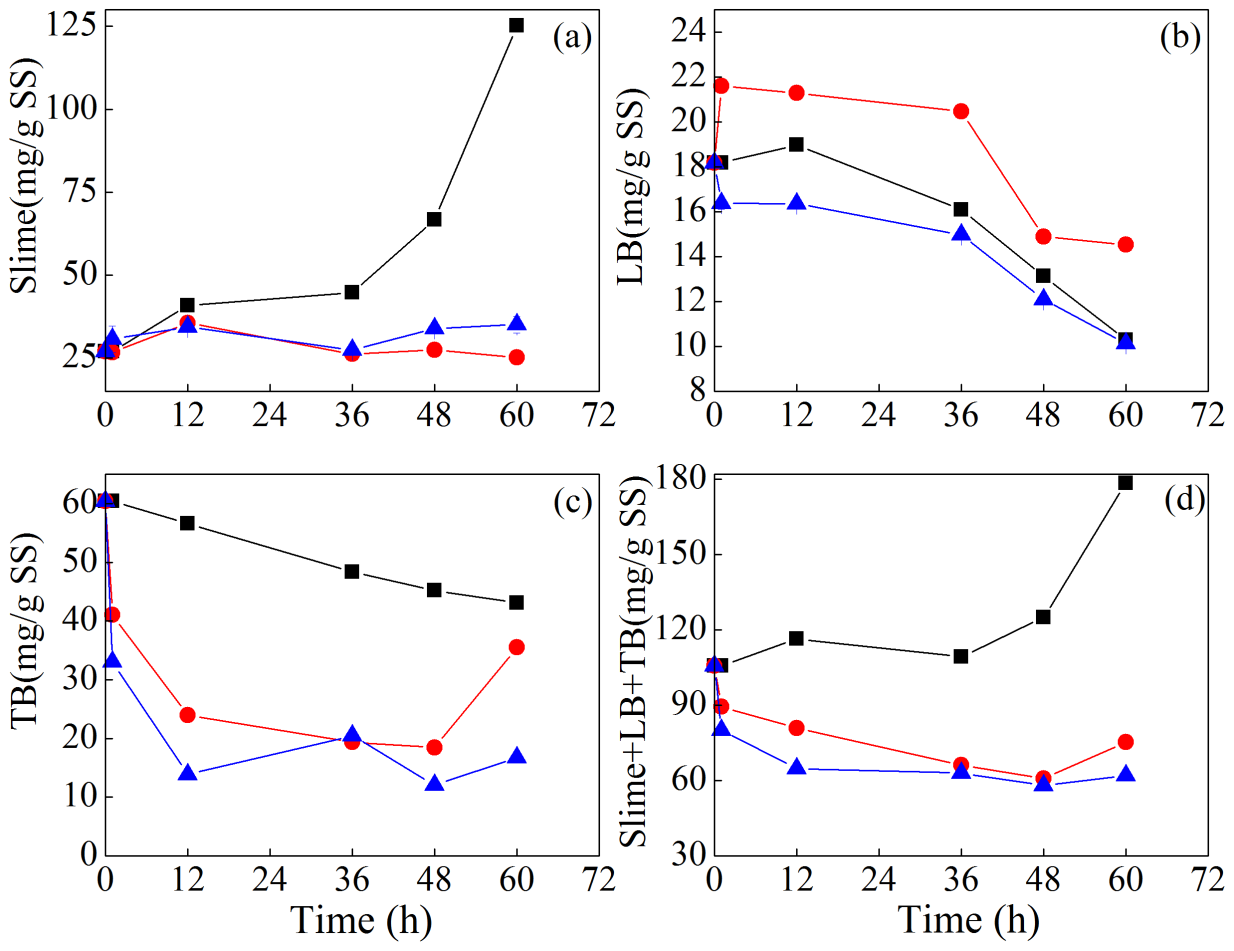
Variation of total EPS content in Slime (a), LB (b), TB (c) and Slime+LB+TB (d) layers during sludge bioleaching process. (▪ CK1, ○CK2, △A.t+A.f).

The change of EPS content in each layer of sludge flocs was shown in Fig. 5. In the original sludge, EPS content in slime, LB-EPS, TB-EPS and Slime+LB+TB (slime-EPS+LB-EPS+TB-EPS) layers were 27.07, 18.17, 60.39 and 105.63 mg/g SS, respectively, and PN/PS was 6.86. It can be seen that the EPS content in the slime layer of CK1 system increased gradually, while EPS content in the slime layer of *A. t*+*A. f* and CK2 systems did not increase during the treatment. The content of LB-EPS in the CK1, CK2 and *A. t*+*A. f* systems decreased during the treatment processes, and TB-EPS content in these systems also gradually decreased during the treatment. Total EPS content (Slime+LB+TB) in CK1 system had a large increase during the treatment, while total EPS content in CK2 and *A. t*+*A. f* systems decreased greatly during the treatment. The total content of EPS in CK1, CK2 and *A. t*+*A. f* systems was 178.52, 75.36 and 61.98 mg/g SS at 60 h, respectively. As set forth, the main characteristics of *A.t*+*A.f* system are low sludge pH and the dominant *Acidithiobacillus* species in the bioleached sludge. Thus, it is presumed that part of EPS was hydrolyzed or degraded in such complex environment, and meanwhile these two autotrophic bacteria probably produced less EPS than heterotrophic microorganisms [37].

### Pearson correlations analyses between sludge CST and protein, polysaccharide, DNA, PN/PS in every layer of sludge flocs

Previous studies revealed that the dewaterability of sludge is influenced by both EPS quantity [16] and the chemical composition of EPS [18]. Pearson correlation analysis between sludge CST and the content of protein, polysaccharide or DNA in each layer of sludge flocs, or PN/PS ratio was performed (see Figure S1), and the results are presented in Table 2. It was found that sludge CST correlated with protein content (R = 0.86469, p<0.0001) in slime-EPS of sludge flocs, while it had no correlation with DNA content (R = 0.39741, p<0.05448), polysaccharide content (R = 0.33576, p = 0.0612) and PN/PS (R = 0.26425, p<0.15171) in slime layer. Thus, sludge dewaterability was affected by protein in the slime layer, and the content increase of protein in slime layer would lead to the deterioration of sludge dewaterability. In turn, the decrease of protein content in slime layer would improve the dewaterability of sludge. The results reported here are consistent with previous studies in that the reduction of protein content in slime layer had significant effect on the dewaterability of sludge [12]. In addition, sludge CST had no correlation with the content of protein (R = 0.21407, p = 0.31516), polysaccharide (R = 0.30185, p = 0.15171) or DNA (R = 0.32089, p = 0.12629), and even PN/PS (R = 0.07999, p = 0.71024) in the LB-EPS layer, and in TB-EPS layer it had correlation with the content of protein (R = 0.64895, p = 0.0006) and polysaccharide (R = 0.69396, p = 0.00017), and no correlation with DNA content (R = 0.59433, p = 0.00219) and PN/PS (R = 0.48251, p = 0.01694). Therefore, the increase of protein and polysaccharide in TB-EPS layer would lead to the deterioration of sludge dewaterability. For the *A. t*+*A. f* system, protein and polysaccharide in TB-EPS layer decreased during bioleaching. One reason may be that these two *Acidithiobacillus* species produced few EPS during the bioleaching, and the other reason might be that protein and polysaccharide were hydrolyzed or degraded at the low pH and high content of Fe^3+^ environment [37]. It was also found that the sludge CST had correlation with protein content (R = 0.74996, p<0.0001) and polysaccharide content (R = 0.94886, p<0.0001) in the Slime+LB+TB layer, while it has no correlation with DNA content (R = 0.4888, p = 0.01536) and PN/PS (R = 0.28393, p = 0.17876) in Slime+LB+TB layer. These results showed that the higher protein and polysaccharide contents in Slime+LB+TB layer, the worse dewaterability of sludge.

**Table 2:** Pearson correlations between sludge CST and the content of protein (a), polysaccharide (b) and DNA (c), or PN/PS (d) from Slime, LB, TB, Slime+LB+TB layer of sludge in the two controls and one bioleaching treatment systems.

Parameters	Protein	polysaccharide	DNA	PN/PS
Slime	R = 0.86469, p<0.0001 (+)	R = 0.33576, p = 0.0612 (×)	R = 0.39141, p = 0.05448 (×)	R = 0.26425, p = 0.21211 (×)
LB-EPS	R = 0.21407, p = 0.31516 (×)	R = 0.30185, p = 0.15171 (×)	R = 0.32089, p = 0.12629 (×)	R = 0.07999, p = 0.71024 (×)
TB-EPS	R = 0.64895, p = 0.0006 (+)	R = 0.69369, p = 0.00017 (+)	R = 0.59448, p = 0.00210 (+)	R = 0.48251, p = 0.01694 (×)
Total EPS	R = 0.74996, p<0.0001 (+)	R = 0.94886, p<0.0001 (+)	R = 0.4880, p = 0.01536 (×)	R = 0.0169, p = 0.17876 (×)

+ Positive correlation; × no correlation.

An analysis of the stratification in this study showed that sludge dewaterability correlated with protein content in slime and both protein and polysaccharide contents in TB-EPS and Slime+LB+TB layers, but not with protein and polysaccharide contents in LB-EPS layer. Although Li and Yang [9] and Novak et al [37] found that sludge dewaterability was correlated with LB-EPS rather than TB-EPS, results reported here demonstrated that the slime and TB-EPS have more important influences on sludge dewaterability than the LB-EPS fraction, which is consistent with that of Yu et al [12]. Therefore, the decrease of protein content in slime and decreases of both protein and polysaccharide contents in TB-EPS and Slime+LB+TB layers were responsible for the sludge dewaterability enhancement in the bioleaching system with the co-inoculation of *A. ferrooxidans* LX5 and *A. thiooxidans* TS6.

### Pearson correlations analyses between sludge CST and sludge pH or the distribution rate of Fe in the solid phase

**Figure 6:**
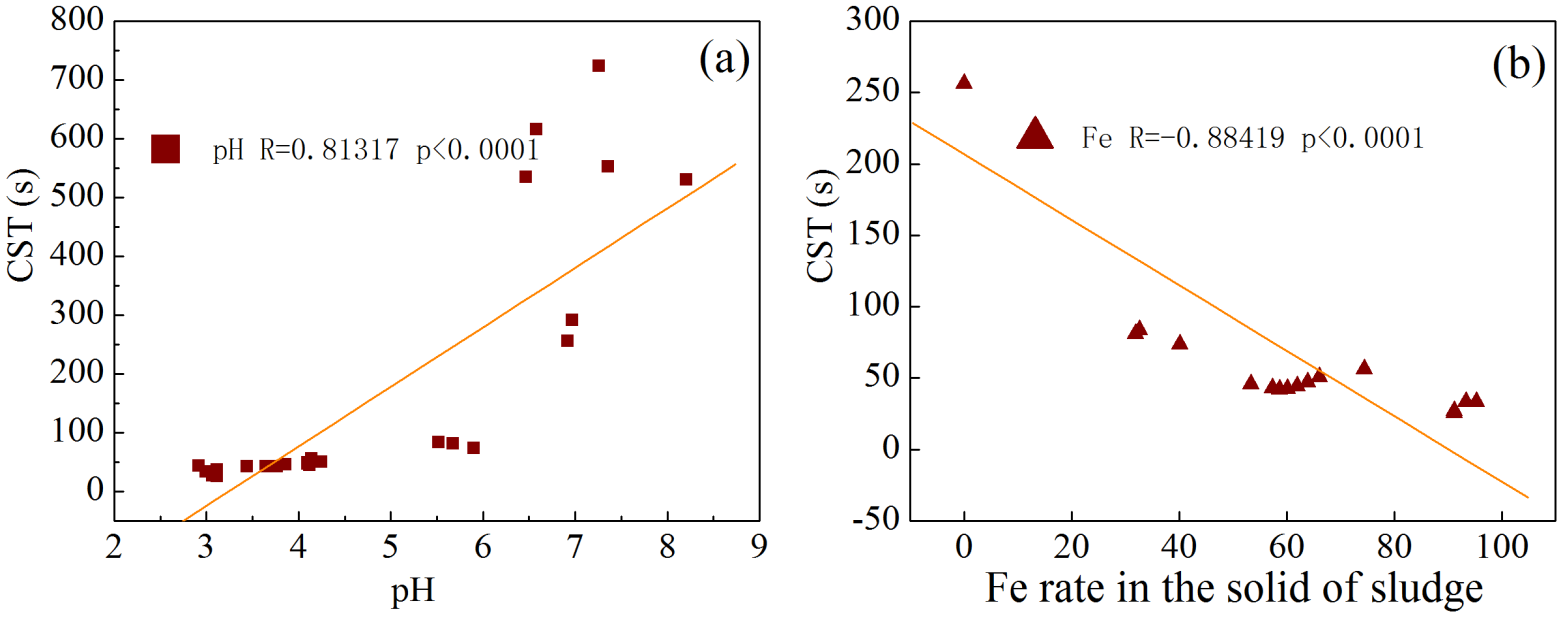
Pearson correlations between sludge CST and sludge pH (a), and the distribution ratio of Fe in the solid phase to the aqueous phase of sludge (b) during sludge bioleaching process.

Literature reported that acid and Fe^3+^ can release the combined water in the sludge particles to free water and thus improve the dewaterability of sludge [1]. Fig. 6 shows Pearson correlation analysis results between sludge CST and sludge pH or the distribution ratio of Fe in the solid phase to the aqueous phase. It was found that sludge CST was positively correlated with sludge pH (R = 0.81317, p<0.0001) and negatively correlated with the distribution ratio of Fe in the solid phase to the aqueous phase (R = 0.88419, p<0.0001). For the bioleaching system, sludge pH was gradually decreased and the distribution ratio of Fe in the solid phase to the aqueous phase was steadily increased. Thus, the low pH and the increasing distribution of Fe in the solid phase were another two factors responsible for the sludge dewaterability improvement. In summary, the growth of *Acidithiobacillus* species during sludge bioleaching process resulted in the decrease of sludge pH, the increasing distribution of Fe in the solid phase, and the decrease of EPS content (mainly including protein and/or polysaccharide) in the slime, TB-EPS, and Slime+LB+TB layers, all of which are helpful for sludge dewaterability enhancement. However, the relative contribution of these factors on sludge dewaterability enhancement cannot be differentiated in the present study.

## Conclusion

The influence of EPS on the sludge dewaterability enhancement during sludge bioleaching was investigated. It was found that the dewaterability of sludge was greatly improved by bioleaching, and sludge CST was reduced from 255.9 s to 25.45 s within 48 h of bioleaching treatment with the co-inoculation of *A. thiooxidans* TS6 and *A. ferrooxidans* LX5, which was obviously better than the control without the inoculation of *Acidithiobacillus* species and the control without either the inoculation of *Acidithiobacillus* species or the addition of energy substrates. Further experiments revealed that the sludge EPS significantly impacted the dewaterability of sludge. Sludge CST had correlation with protein content in slime and both protein and polysaccharide contents in TB-EPS and Slime+LB+TB layers, and the decrease of protein content in slime and decreases of both protein and polysaccharide contents in TB-EPS and Slime+LB+TB layers improved sludge dewaterability during sludge bioleaching process. Moreover, the low pH and the increasing distribution of Fe in the solid phase were another two factors responsible for the improvement of sludge dewaterability during bioleaching. Further research is needed to explore the possibility of reducing the protein and polysaccharide contents in sludge EPS through combining some chemical and/or physical methods with bioleaching to further improve sludge dewaterability during sludge bioleaching treatment.

## Supporting Information

Figure S1 **Pearson correlations between CST and the content of protein (a), polysaccharide (b), DNA (c), or PN/PS (d) from Slime, LB, TB, and Slime+LB+TB layer of sludge in in the two controls and one bioleaching treatment systems (doc).** This material is available free of charge via the Internet at http://www.plosone.org.
(DOC)
