# Supplementary material for: Influences of Extracellular Polymeric Substances on the Dewaterability of Sewage Sludge during Bioleaching
Source: PLoS One. 2014 Jul 22;9(7):e102688. doi: 10.1371/journal.pone.0102688 (PMC4106846; doi:10.1371/journal.pone.0102688)
Supplement: Figure S1 — Pearson correlations between CST and the content of protein (a), polysaccharide (b), DNA (c), or PN/PS (d) from Slime, LB, TB, and Slime+LB+TB layer of sludge in in the two controls and one bioleaching treatment systems (doc). This material is available free of charge via the Internet at http://www.plosone.org. (DOC) [file pone.0102688.s001.doc]

**Supplemental materials** (This figure is equal to the table 2 in the manuscript)**:**

Figure S1 Pearson correlations between CST and the content of protein (a), polysaccharide (b), DNA (c) , or PN/PS (d) from Slime, LB, TB, Slime+LB+TB layer of sludge in in the two controls and one bioleaching treatment systems.
